# Investigating 4D respiratory cone-beam CT imaging for thoracic interventions on robotic C-arm systems: a deformable phantom study

**DOI:** 10.1007/s13246-024-01491-0

**Published:** 2024-10-24

**Authors:** Tess Reynolds, Owen Dillon, Yiqun Ma, Nicholas Hindley, J. Webster Stayman, Magdalena Bazalova-Carter

**Affiliations:** 1 University of Sydney, Sydney, NSW Australia; 2 Johns Hopkins University, Baltimore, MD USA; 3 University of Victoria, Victoria, BC Canada

**Keywords:** Cone-beam CT, Respiratory, 4DCBCT, Interventional imaging

## Abstract

Increasingly, interventional thoracic workflows utilize cone-beam CT (CBCT) to improve navigational and diagnostic yield. Here, we investigate the feasibility of implementing free-breathing 4D respiratory CBCT for motion mitigated imaging in patients unable to perform a breath-hold or without suspending mechanical ventilation during thoracic interventions. Circular 4D respiratory CBCT imaging trajectories were implemented on a clinical robotic CBCT system using additional real-time control hardware. The circular trajectories consisted of 1 × 360° circle at 0° tilt with fixed gantry velocities of 2°/s, 10°/s, and 20°/s. The imaging target was an in-house developed anthropomorphic breathing thorax phantom with deformable lungs and 3D-printed imaging targets. The phantom was programmed to reproduce 3 patient-measured breathing traces. Following image acquisition, projections were retrospectively binned into ten respiratory phases and reconstructed using filtered back projection, model-based, and iterative motion compensated algorithms. A conventional circular acquisition on the system of the free-breathing phantom was used as comparator. Edge Response Width (ERW) of the imaging target boundaries and Contrast-to-Noise Ratio (CNR) were used for image quality quantification. All acquisitions across all traces considered displayed visual evidence of motion blurring, and this was reflected in the quantitative measurements. Additionally, all the 4D respiratory acquisitions displayed a lower contrast compared to the conventional acquisitions for all three traces considered. Overall, the current implementation of 4D respiratory CBCT explored in this study with various gantry velocities combined with motion compensated algorithms improved image sharpness for the slower gantry rotations considered (2°/s and 10°/s) compared to conventional acquisitions over a variety of patient traces.

## Introduction

Cone Beam Computed Tomography (CBCT) imaging is increasingly being integrated into interventional workflows. Focusing specifically on thoracic interventions, CBCT imaging is primarily used for procedural guidance/navigation and verification. Recent examples include, CBCT-guided video assisted thoracic surgery [1], CBCT-guided diagnostic bronchoscopy [2], monitoring of ablation zones surrounding lung [3, 4] and liver tumors, diagnosing peripheral pulmonary lesions [5], and providing navigation during biopsies [6, 7] and lobectomies [8]. One of the challenges faced by current interventional thoracic CBCT acquisitions is patient respiratory motion.

Currently, patient respiratory motion is minimized by suspending mechanical ventilation during imaging [9, 10]. Suspending ventilation, however, restricts imaging to capture only a single respiratory phase (e.g., inhale or exhale), eliminating the possibility of gaining full 4D respiratory motion information. Additionally, not all thoracic interventions require the patient to be ventilated. In some liver interventions, for example, patient motion is minimized by imaging during a breath-hold [11]. However, not all patients are able to perform a breath-hold. As such, there remains an unmet need to provide free-breathing respiratory CBCT imaging acquisitions for use during thoracic interventions.

One pathway to facilitate free-breathing respiratory CBCT imaging in the interventional room is to leverage the established body of work surrounding pre-treatment CBCT imaging during thoracic radiotherapy [12]. For example, recent advances in acquisition techniques have enabled the ability to produce fast, high quality, and low-dose respiratory correlated CBCT images on clinical imaging hardware [13].

**Fig. 1:**
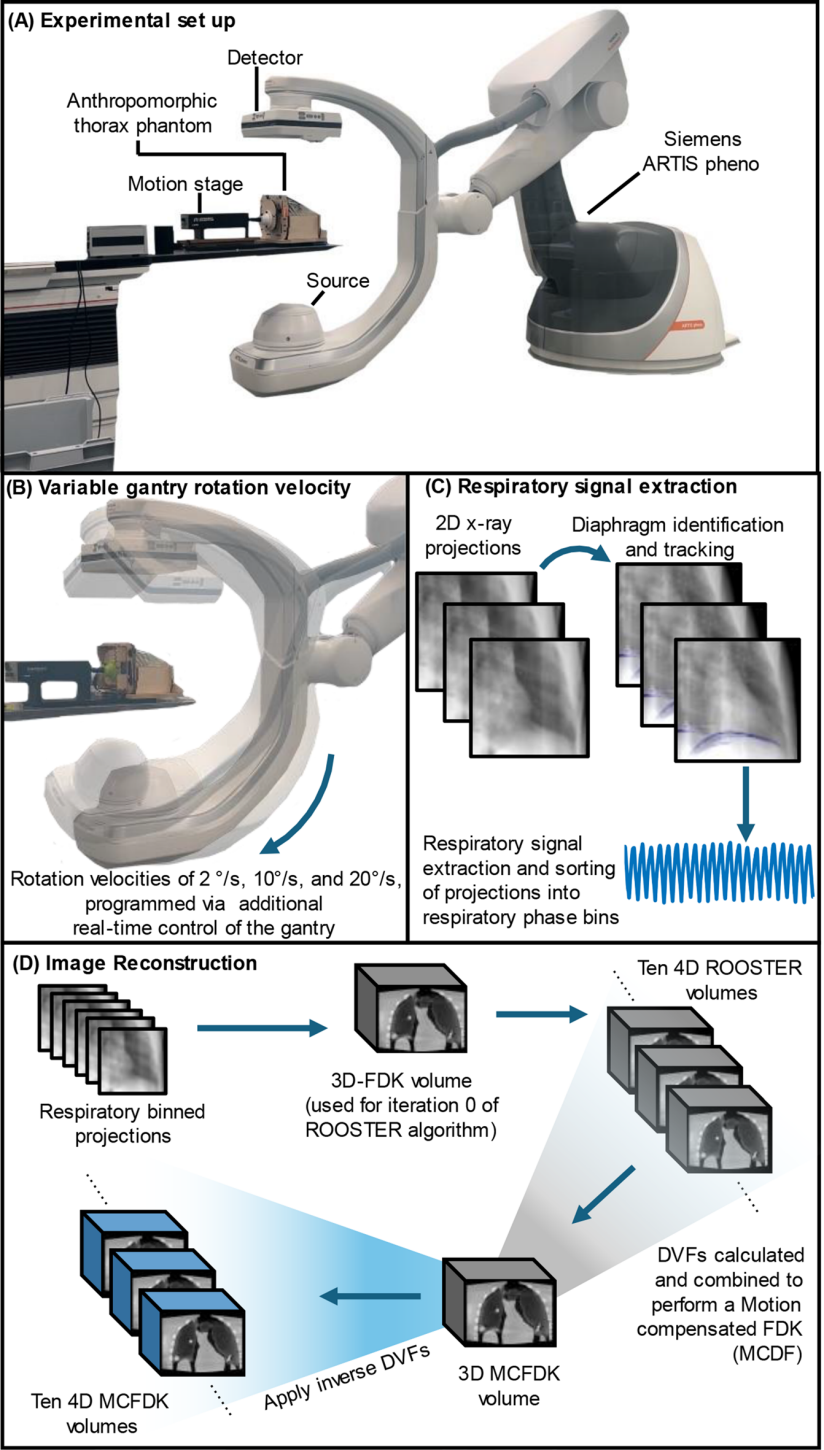
Overview of the implementation of the proposed 4D Respiratory CBCT imaging on a clinical robotic CBCT imaging system. **A** Experimental set up, **B** circular acquisitions with various gantry velocities, **C** respiratory signal extraction, and **D** image reconstruction

The objective of this study is to investigate the feasibility of implementing free-breathing 4D respiratory CBCT for motion-mitigated imaging within a hybrid CBCT operating room. Specifically, we target patients undergoing interventional thoracic procedures that are unable to perform a breath-hold or without suspending mechanical ventilation during imaging. We hypothesize that free-breathing 4D respiratory CBCT imaging can be implemented on a clinical robotic floor-mounted CBCT imaging system within a hybrid CBCT operating room for motion-mitigated respiratory imaging. Our proposed free-breathing 4D respiratory CBCT imaging leverages acquisition and reconstruction approaches from our previous studies in advancing pre-treatment radiotherapy 4D CBCT imaging on linear accelerators. To demonstrate the potential of the proposed CBCT imaging method to reduce respiratory motion during thoracic interventions, we implement 4D respiratory CBCT acquisitions on a clinical robotic floor-mounted CBCT imaging system, using additional real-time control of the gantry rotation, acquiring images of an anthropomorphic breathing thorax phantom with deformable lungs containing multiple imaging targets and compare to conventional 3D thoracic CBCT imaging currently used clinically, Fig. 1.

## Methods

Circular CBCT imaging trajectories were implemented on a clinical floor mounted robotic CBCT imaging system (Siemens ARTIS pheno, Siemens Healthineers GmbH, Erlangen, Germany). Control of the gantry during the 4D respiratory CBCT acquisitions (i.e., gantry rotation velocity) was provided through additional real-time control hardware provided by Siemens, known as the Siemens Test Automation Control System (TACS). As comparator, conventional CBCT acquisitions were acquired with no motion, representing a perfect breath-hold or suspension of ventilation, and with motion, representing free-breathing conditions. An in-house developed programmable anthropomorphic breathing thorax phantom with deformable lungs and 3D-printed imaging targets [14] was imaged.

### 4D respiratory CBCT acquisition

The 4D respiratory CBCT acquisitions considered had circular trajectories spanning 360° with fixed gantry rotation velocities of 2°/s, 10°/s, and 20°/s. The gantry rotation velocities were selected to investigate the trade-off between image quality, imaging dose, and scan time when conducting 4D respiratory CBCT in the interventional room. Gantry velocities between 20°/s and 45°/s (current maximum clinical gantry rotation velocity) could not be investigated as the maximum gantry rotation velocity is restricted to 20°/s when the gantry is controlled via the TACS. During the circular respiratory CBCT acquisitions, 2D X-ray projections were acquired at 90 kV and 0.5 mAs. The frame rate of each of the acquisitions was determined by the gantry rotation velocity. Specifically for the 2°/s, 10°/s, and 20°/s gantry rotation velocity acquisitions, projection rates of 7.5, 15, and 30 frames/second were used, resulting in approximately 1450, 600, 540, total projections respectively. To facilitate 4D CBCT reconstruction, the 2D X-ray projections were retrospectively sorted into 10 respiratory phase bins (Sect. “Respiratory signal extraction”) and volumetric images generated via motion compensated reconstruction algorithms (Sect. “Motion compensated reconstruction”).

### Conventional acquisition

The comparator imaging acquisition method was an in-built protocol available on the Siemens ARTIS pheno named 5s Dyna CT Body (Siemens Healthineers, GmbH), referred to throughout as the conventional acquisition. The conventional acquisition is a circular imaging trajectory spanning 200°, with gantry velocity of up to 40°/s, acquiring 397 2D X-ray projections at 90 kV and 0.5 mAs within a 5 s scan time. The conventional acquisitions were reconstructed using the in-built algorithms provided on the Siemens ARTIS pheno.

### Anthropomorphic breathing phantom and respiratory traces

**Fig. 2:**
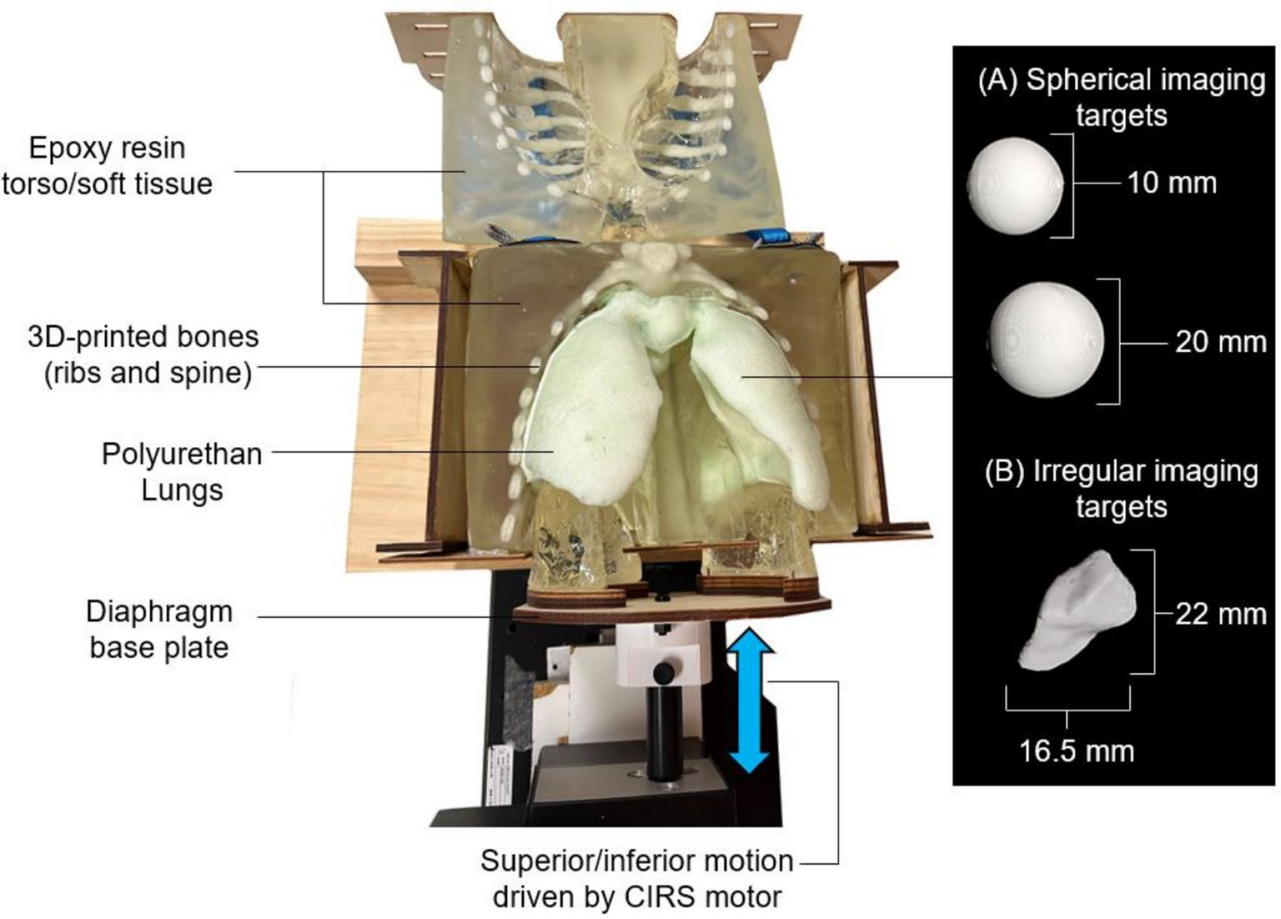
Anthropomorphic thorax phantom with deformable polyurethan lungs embedded with 3D-printed polylactic acid (PLA) **A** spherical and **B** irregular imaging targets

The imaging target was an in-house developed programmable anthropomorphic breathing thorax phantom with deformable lungs [14], referred to throughout simply as the phantom. To enable further control and customization of the breathing motion, the phantom was modified from its original form to allow integration with a CIRS Dynamic Thorax motor (Sun Nuclear Corporation) via a 3D-printed polylactic acid (PLA) connector attached to the diaphragm plate, Fig. 2. Additionally, to increase the complexity of the phantom, a new set of deformable foam lungs were produced embedded with varying 3D-printed PLA inserts as imaging targets. The lungs contained 3D-printed PLA spherical and irregular imaging targets, shown prior to insertion in Fig. 2 (A) and (B) and within the deformable lungs in Fig. 4. The smaller (diameter 10 mm) spherical imaging target in the middle right lung is referred to as Sphere 1, while the larger (diameter 20 mm) spherical imaging target in the lower right lung is referred to as Sphere 2. The shape of the irregular imaging target was taken from a thoracic cancer patient planning target volume contour.

**Fig. 3:**
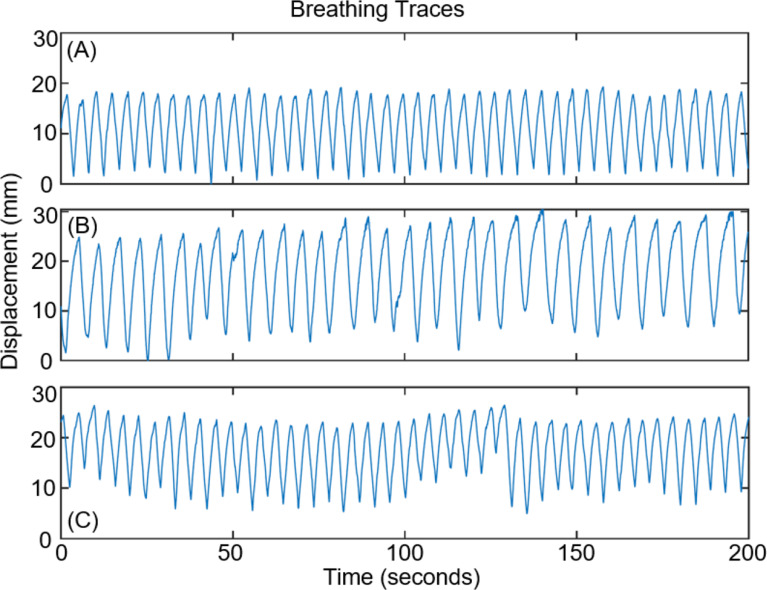
First 200 s of the breathing traces programmed into the phantom. **A** patient trace 1, **B** patient trace 2 and **C** patient trace 3

The phantom was programmed to reproduce the motion of 3 patient measured breathing traces. The three patient measured breathing traces were sourced from the Virginia Commonwealth University (VCU) breathing training database [15]. The VCU database contains a total of 644 breathing traces acquired from 55 patients. To simulate a variety of breathing variability and irregularity during this study, the breathing traces were stratified based on the displacement root mean square error (RMSE). Sorting the traces by RMSE percentile, 13 traces were identified representing the 5th, 10th, 20th, 25th, 30th, 40th, 50th, 60th, 70th, 75th, 80th, 90th and 95th percentiles respectively, as has been reported in our previous respiratory-based imaging technology development investigations [16]. From these 13 traces, 3 were selected representing the 5th, 30th, and 50th percentiles, providing a variety of breathing variability and irregularity to be investigated, while also ensuring that the traces could be accurately and consistently reproduced with the phantom and motion stage used in the study. Patient trace 1 had a mean amplitude of 16.1 mm, mean breathing period of 4.5 s and RMSE of 0.18. Patient trace 2 had a mean amplitude of 21.6 mm, mean breathing period of 6.5 s, and RMSE of 0.37. Patient trace 3 had a mean amplitude of 15.4 mm, mean breathing period of 4.5 s, RMSE of 0.52. The first 200 s of each of the breathing traces used in this study are displayed in Fig. 3.

### Image reconstruction

#### Geometric calibration

The 4D respiratory CBCT acquisitions required an additional post-processing step, specifically an online geometric calibration, prior to reconstruction to ensure high quality 3D image reconstruction was possible. An online geometric calibration step was required due to low reducibility of imaging trajectories implemented with the TACS. Here, we implemented a fiducial-based online geometric calibration method that involved placing 12 spherical fiducials on the exterior of the programmable anthropomorphic breathing thorax phantom. Full details of the online geometric calibration method are provided elsewhere [17].

#### Respiratory signal extraction

To enable phase binning of the 4D respiratory CBCT acquisitions, a respiratory signal was extracted from each projection using a direct diaphragm algorithm described previously [18], Fig. 1(C). Briefly, the diaphragm is segmented by searching over each coronal slice according to the following criteria. First, the optimal segment should span the largest distance in the left/right direction. Second, the outermost points of the optimal segment will extend the furthest from the image center. Third, the optimal segment will exhibit negative curvature. The optimal segment is then regularized by a smoothing spline in both the left/right and anterior/posterior directions and forward-projected to 2D projection space over a full 360° rotation. On each projection, the diaphragm is then tracked via a modified maximum gradient algorithm. In particular, a vertical difference image is calculated for each image and the superior/inferior displacement of the diaphragm is measured as for which the pixel intensity difference is maximized over the projected 2D diaphragm mask. The resulting respiratory signal was then used to determine the respiratory phase for each projection.

#### Motion compensated reconstruction

Following geometric calibration, all 4D respiratory CBCT reconstructions were performed using a combination of methods implemented in the Reconstruction Tool Kit (RTK) [19]. An initial reconstruction was computed using the 3D Feldkamp-Davis-Kress (FDK) algorithm [20] in which all projections are filtered and backprojected, resulting in an image with motion blur around moving structures. The 3D FDK image is used as iteration 0 of the RecOnstructiOn method using Spatial and Temporal Regularization (ROOSTER) algorithm [21], which employs Total Variation regularization over both space (between voxel) and time (between each 3D frame of the 4D image). Each 3D frame of the 4D ROOSTER image is deformably registered to the peak inhale 3D frame using Elastix [22]. The resulting Deformable Vector Fields (DVFs) are then combined to perform a Motion Compensated FDK (MCFDK) reconstruction using all acquired projections of the peak inhale phase. The peak inhale MCFDK image is then deformed using the inverse DVFs to create the full 4D MCFDK image, Fig. 1 (D).

### Reconstructed image quality analysis

The image quality of all the reconstructed volumes generated in this study were qualitatively assessed via visual inspection, and quantitatively assessed using two image quality metrics. Namely, the Edge Response Width (ERW) and Contrast-to-Noise Ratio (CNR).

**Fig. 4:**
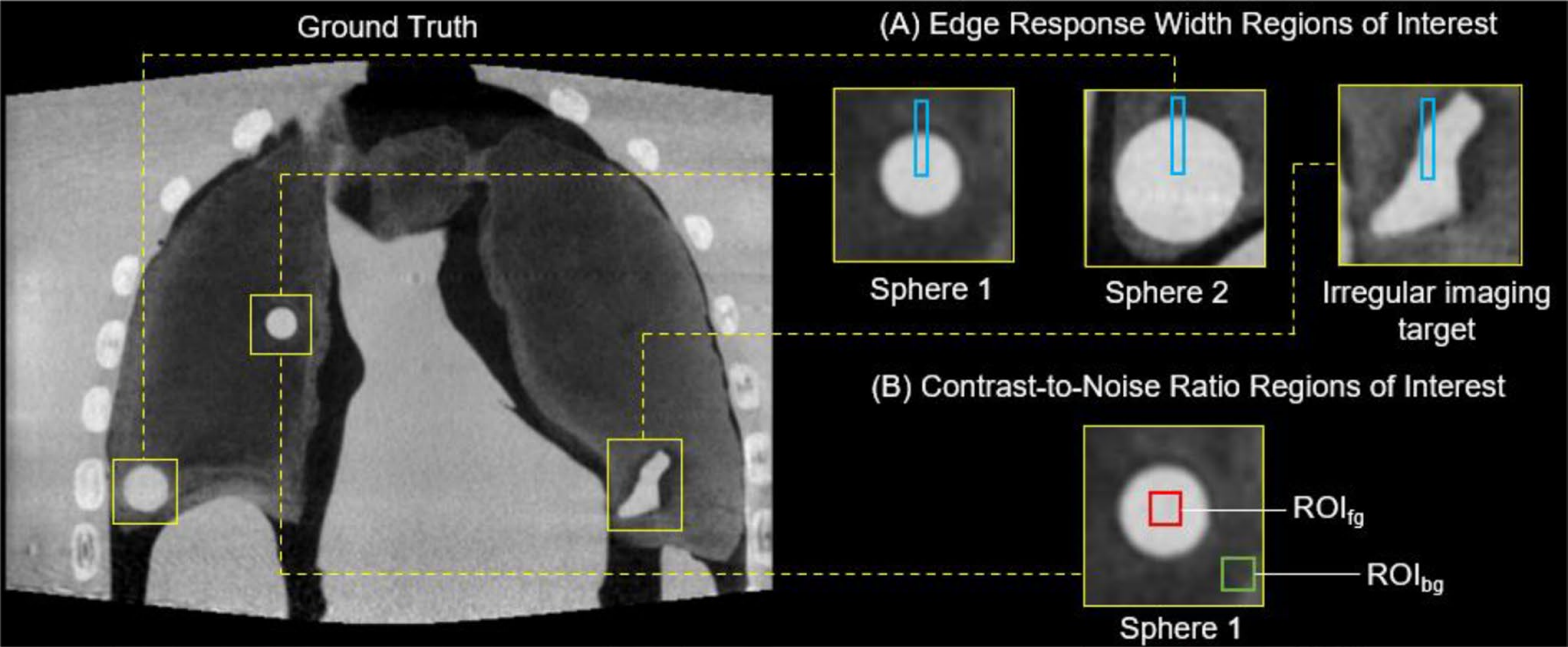
Locations of the regions of interest for calculating **A** the Edge Response Width and **B** contrast-to-noise ratio of the acquisitions considered in this study

Here, the ERW provides an indication of the sharpness of the edges of the three imaging targets and is calculated across the primary direction of motion (superior/inferior) [23]. The ERW is calculated by examining linear profiles across the imaging target/lung boundary for each of the three imaging target inserts, Fig. 4A. The ERW is defined as the width, measured in millimeters, between the 25% and 75% change in intensity across the linear profile. A smaller ERW value indicates a sharper, more clearly defined boundary between two distinct regions.

The CNR was calculated using:

1 $$\:CNR=\frac{|{\mu}_{ROIfg}-{\mu}_{ROIbg}|}{\sigma}$$

Where *µ*_*ROIfg*_ and *µ*_*ROIbg*_ were the mean pixel values of the foreground and background 10 × 10 mm^2^ regions of interest respectively, as shown in Fig. 4 (B), and *σ* was the standard deviation within the background region of interest. Note that CNR is dimensionless but can be understood as the number of standard deviations separating the foreground and background mean values. The CNR was calculated over sphere 1 only to ensure an even background region, with the other inserts residing close to the lung/air boundary and diaphragm.

## Results

### CBCT images

**Fig. 5:**
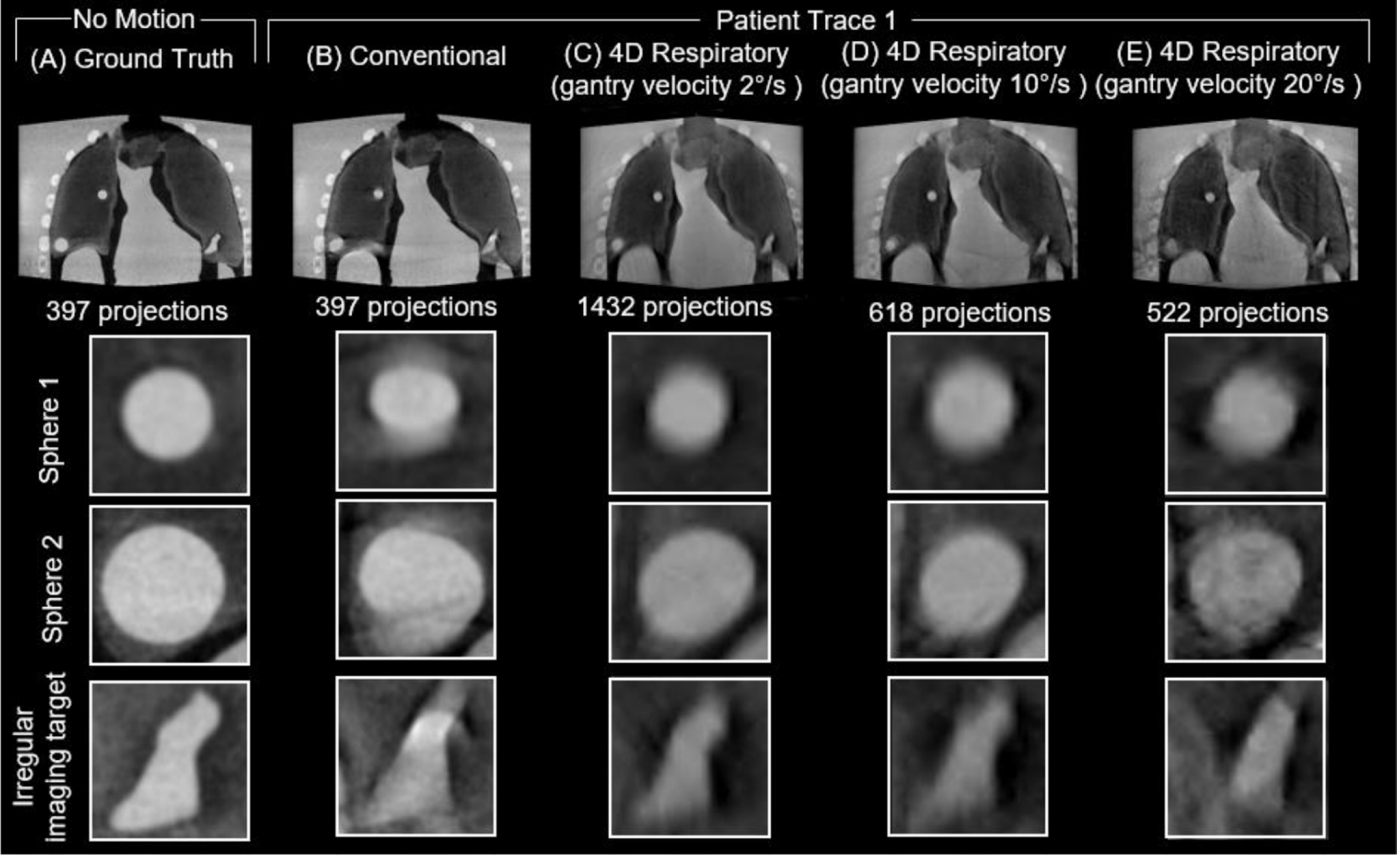
Reconstructed images from **A** ground truth, **B** conventional and respiratory phase 6 of the 4D respiratory acquisitions with gantry velocity of **C** 2°/s, **D** 10°/s, and **E** 20 °/s for Patient Trace 1. Close ups show the 3D-printed imaging targets

**Fig. 6:**
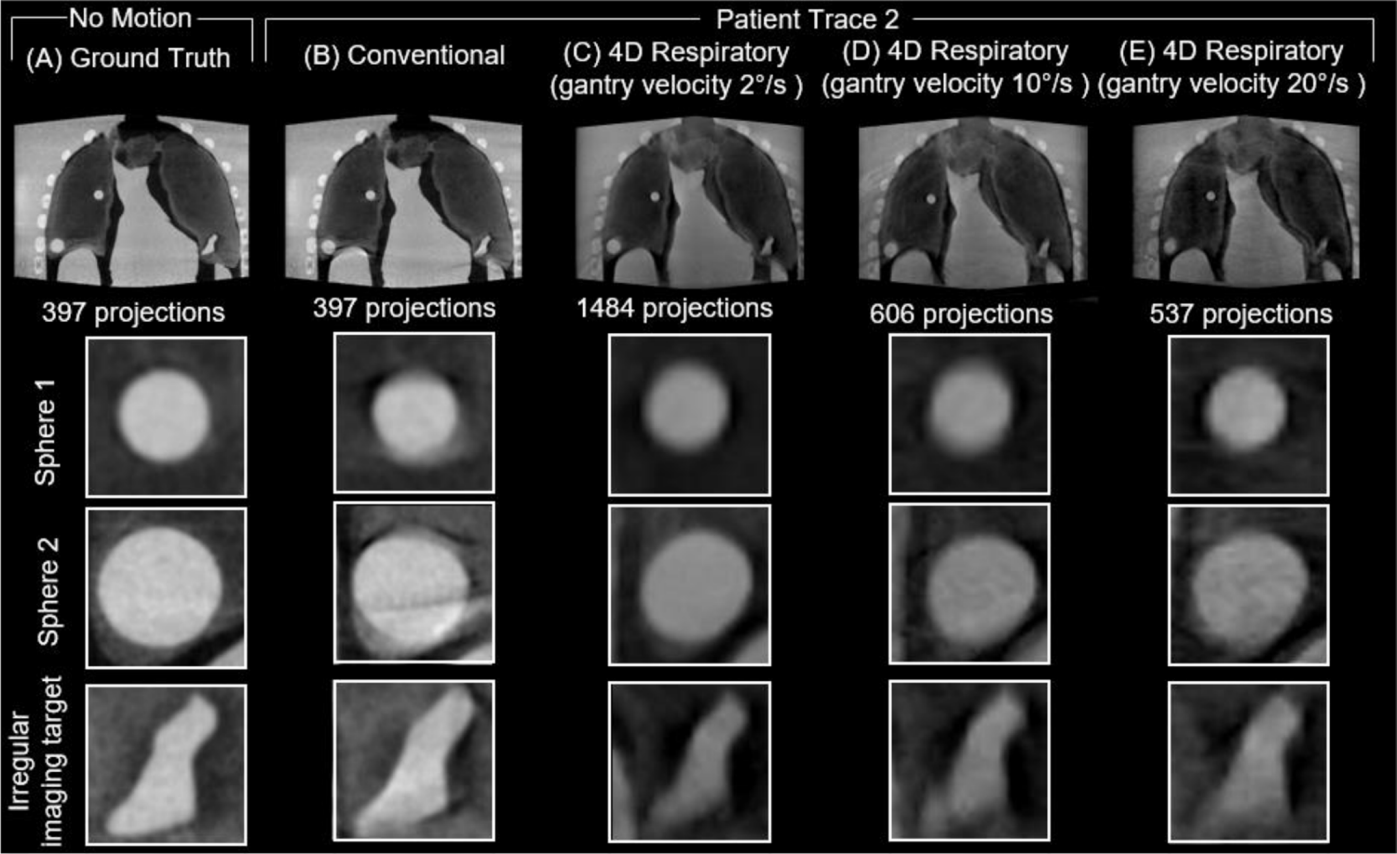
Reconstructed images from **A** ground truth, **B** conventional and respiratory phase 6 of the 4D respiratory acquisitions with gantry velocity of **C** 2°/s, **D** 10°/s, and **E** 20 °/s for Patient Trace 2. Close ups show the 3D-printed imaging targets

**Fig. 7:**
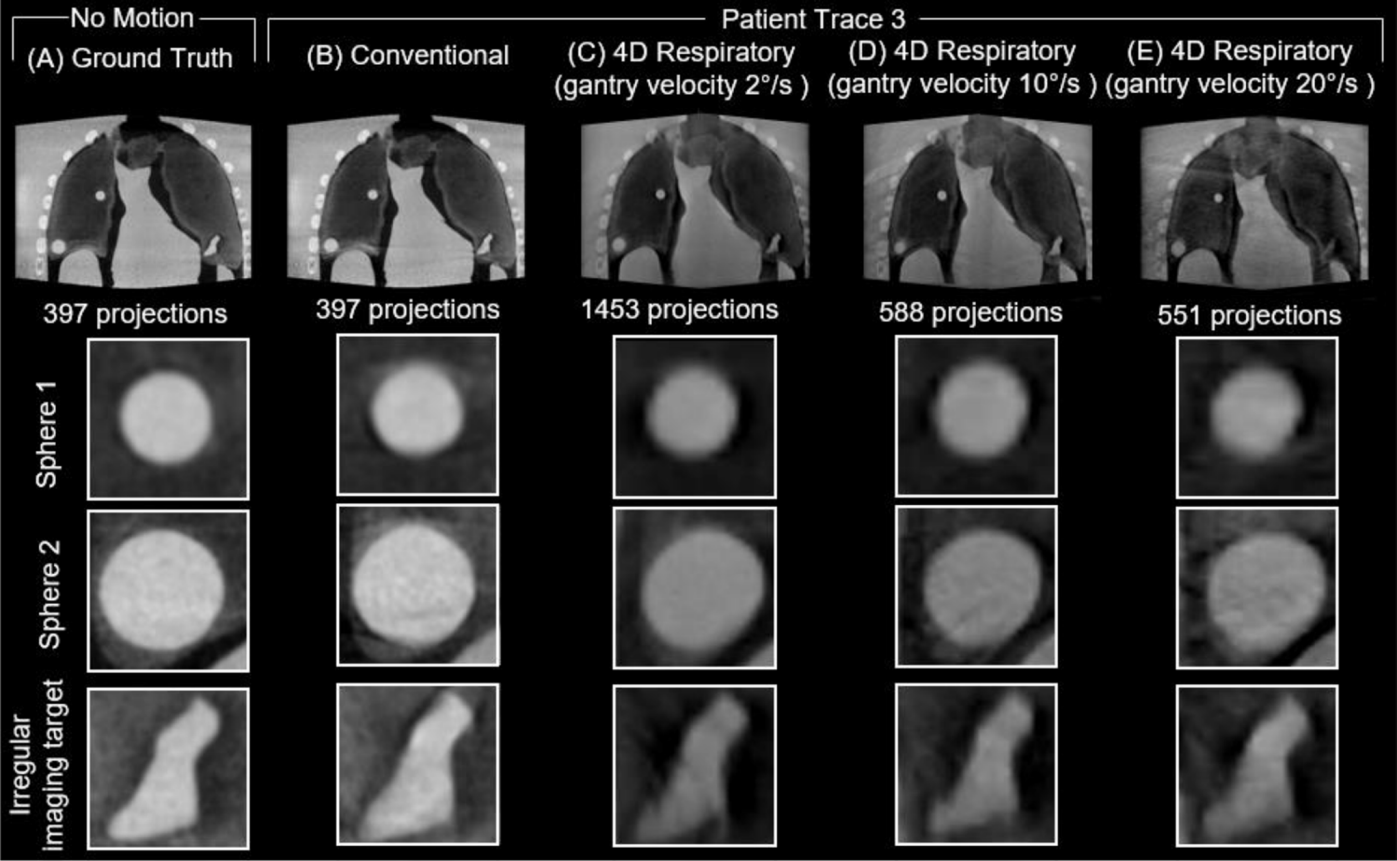
Reconstructed images from **A** ground truth, **B** conventional and respiratory phase 6 of the 4D respiratory acquisitions with gantry velocity of **C** 2°/s, **D** 10°/s, and **E** 20 °/s for Patient Trace 3. Close ups show the 3D-printed imaging targets

A sample of the reconstructed 3D images of the anthropomorphic breathing thorax phantom with no motion (ground truth) and the 3 patient traces for the conventional and three 4D respiratory acquisitions with varying gantry velocity are provided in Figs. 5, 6 and 7, respectively. For each acquisition, an axial slice displaying the entire phantom volume is provided, along with close ups of each of the three 3D-printed inserts within the deformable lungs. All volumes have the same window width (0.018 mm^-1^) and window level (0.012 mm^-1^) settings.

Compared to the ground truth, all acquisitions across all traces considered display visual evidence of blurring and artifacts due to the programmed respiratory motion in the phantom. Additionally, all the 4D respiratory acquisitions display a lower contrast compared to the conventional acquisitions for all three traces considered.

The most noticeable visual differences between the conventional and 4D respiratory acquisitions can be observed in patient trace 1 and 3. In the conventional acquisition images of patient trace 1 and 3, there are clearly observable motion artifacts around both sphere 1 and sphere 2, which are resolved in all three 4D respiratory acquisitions. Comparatively, there are minimal observable motion artifacts in either the conventional or 4D respiratory acquisitions in patient trace 2. As the number of projections in the 4D respiratory acquisitions decreased with increasing gantry velocity, there was an observable increase in the amount of noise in the reconstructed images across all three traces.

### Quantitative image assessment

**Fig. 8:**
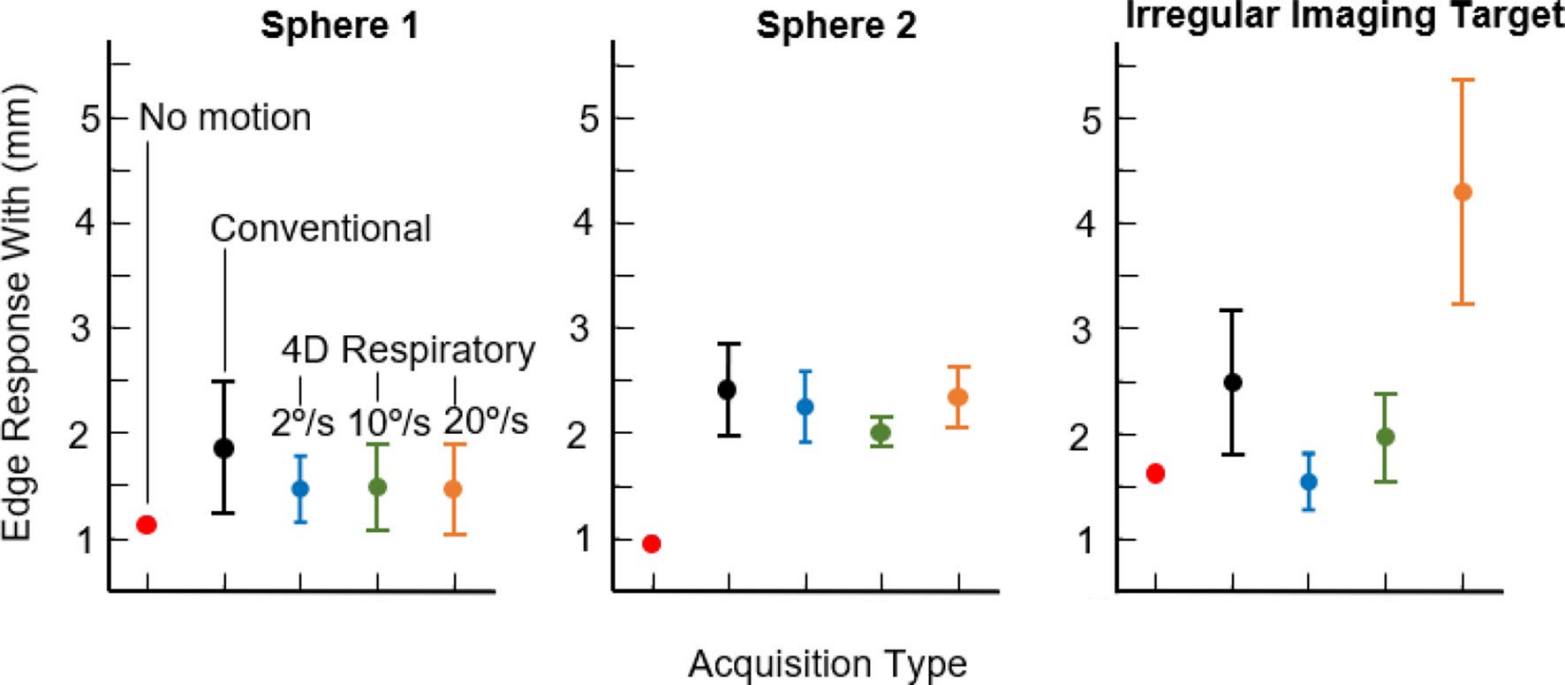
The edge response widths across **A** Sphere 1, **B** Sphere 2, and **C** the irregular imaging target, for the conventional (black) and 4D respiratory acquisitions with gantry velocity of 2°/s (blue) 10°/s (green) and 20 °/s (orange) compared to the ground truth (red), for all traces

The image quality of each volume generated in this study was quantified via ERW, a measure of imaging target edge sharpness, and CNR. The average and standard deviation of the ERW measurements across the three 3D-printed imaging targets for all acquisitions and traces are provided in Fig. 8. Comparing the ERW measurements between the conventional and 4D respiratory acquisitions with 2°/s, 10°/s, and 20°/s across all 3D-printed imaging targets as two sample t-tests produces p-values of 0.046, 0.044, and 0.35 respectively. This indicates that the 2°/s and 10°/s 4D respiratory acquisitions produce a significant reduction in ERW compared to the conventional scan, which is confirmed via visual inspection of the reconstructed images showing improved image sharpness across the 3D-printed imaging targets. There is no significant improvement in the ERW measurements when comparing between the respective 4D respiratory acquisitions. Specifically for the irregular tumour insert, there were large variations in the ERW from the 4D respiratory acquisitions with 20°/s gantry rotation velocity compared to the conventional acquisition, indicating a reduction in the image sharpness.

**Fig. 9:**
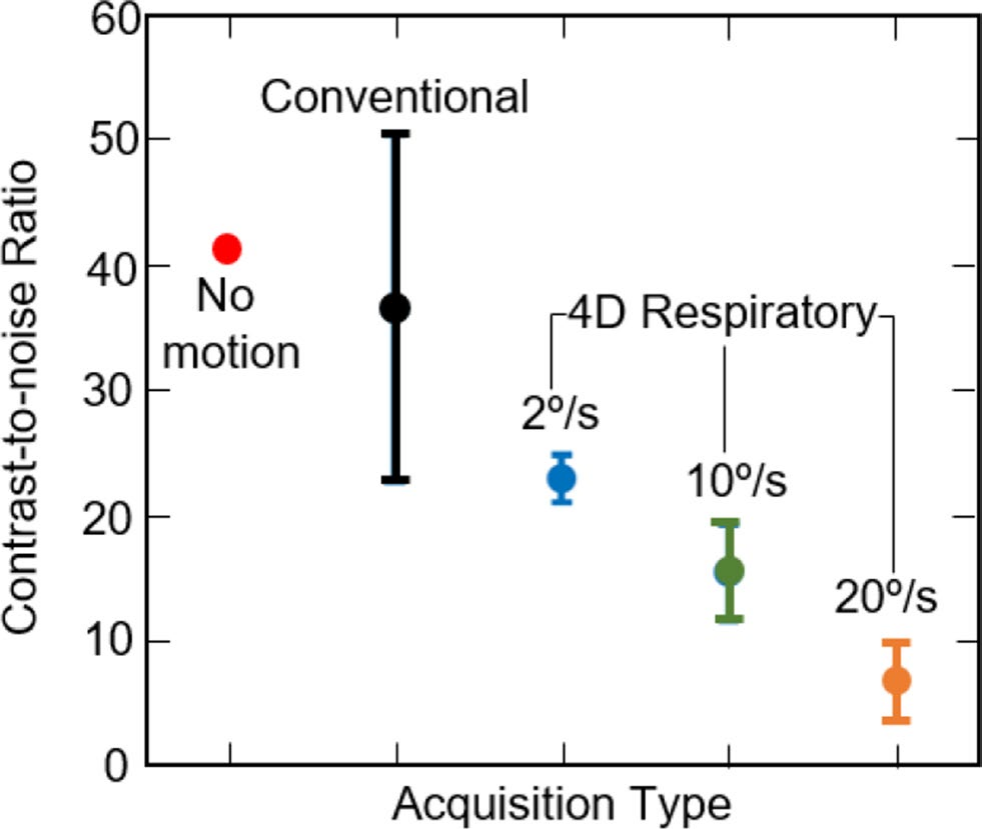
The Contrast-to-Noise ratio for the conventional (black) and 4D respiratory acquisitions with gantry velocity of 2°/s (blue) 10°/s (green) and 20 °/s (orange) compared to the ground truth (red), for all traces

The average and standard deviation of the CNR for all the acquisitions considered across all traces are provided in Fig. 9. While there is a much larger spread in the CNR for the conventional acquisitions across all traces, the CNR of 4D respiratory acquisitions decreases with decreasing number of projections (i.e., increasing gantry velocity). This again aligns with the visual observations above.

## Discussion

4D CBCT has become a key technology for identifying and tracking moving targets within the lung and liver, with most notable impact to date occurring within radiation therapy [24]. Comparatively, respiratory motion remains an elusive challenge for interventional procedures, despite the growing demand [25] and success in mitigating cardiac motion during imaging [26–29], for example. One approach to address the challenge of respiratory motion during interventional imaging and image-guided procedures, as taken in this study, is to leverage the advances made within radiation therapy for 4D CBCT and apply them to robotic CBCT imaging systems that facilitate procedures in interventional radiology, pulmonology, and image-guided surgery. Other recent works have attempted to address the challenge of respiratory motion during imaging and image-guided procedures by integrating additional hardware (i.e. optical and electromagnetic tracking methods [30, 31]) and developing advanced algorithmic [32, 33] and deep learning approaches [34]. To date, these proposed approaches have shown initial success but have not seen widespread clinical uptake. Space is a premium in current interventional and hybrid operating rooms, and as such, solutions that require the integration of additional hardware face potential challenges to achieving clinical integration. Comparatively, time is the other premium in interventional and hybrid operating rooms and so software and algorithmic approaches must be able to execute and deliver results within seconds to enable real-time guidance and decision making.

This study investigated the feasibility of implementing free-breathing 4D respiratory CBCT with a range of fixed gantry velocities for motion-mitigated imaging in patients unable to perform a breath-hold or without suspending mechanical ventilation during thoracic interventions. Overall, the current implementation of 4D respiratory CBCT explored in this study with various gantry velocities combined with motion compensated algorithms produce improved image sharpness for the slower gantry rotation velocities considered (2°/s, 10°/s) compared to conventional acquisitions over a variety of patient traces. This motivates further work to deliver a faster and robust 4D respiratory CBCT imaging technique that can be used in interventional radiology, pulmonology, and image-guided surgery.

There were a number of limitations present in the current study. Firstly, the deformable phantom contained simplified anatomy. For example, within the lungs of the phantom, the only anatomical features were solid 3D-printed imaging targets that were not deformable. As such, the imaging targets experienced rigid movement within the deformable foam lungs. However, designing and manufacturing deformable thorax phantoms that accurately mimic human physiology and respiratory motion is challenging and represents its own body of work beyond what is presented in this study. At present, despite the prevalence of rigid motion thorax phantoms available for applications in radiology and radiotherapy [35], there are not currently any commercially available deformable thorax phantoms. As such, researchers must rely on in-house developed phantoms in the preliminary investigations of novel imaging approaches. The challenge of developing suitable phantoms to evaluate and benchmark advances in medical imaging technologies remains unmet within the field, as highlighted by a recent perspective article in Communications Engineering [36]. This is especially important for replicating complex motion, such as respiration, which is central to refining and understanding limits of motion-mitigation imaging technologies prior to moving to clinical trials and implementation. Secondly, to facilitate the phase binning in the 4D respiratory CBCT acquisitions without introducing additional hardware into the imaging workflow not native to interventional procedures (e.g., Real-time Position Management system), a diaphragm tracking algorithm was implemented. In this study, the diaphragm tracking algorithm had to be implemented retrospectively as access to real-time projection and geometric calibration data was not available. Implementing real-time diaphragm tracking would be feasible in future 4D respiratory CBCT acquisition with additional vendor support. Thirdly, the only way to implement novel CBCT imaging trajectories in real-time on clinical robotic CBCT imaging systems is with additional software or hardware control provided by the vendor, introducing a number of additional limitations as previously outlined [37–40]. Specifically for this study, the limitations pertain to the maximum velocity of the gantry rotation when using the TACS and the quality of the projection data available for image reconstruction. The gantry rotation velocity is limited to only 20°/s when the TACS is used to program and control the C-arm movements, compared to up to 45°/s in clinical use. As such, only gantry rotations between 0–20°/s can be investigated. Further, the system is currently not designed to enable arbitrary trajectory 3D imaging acquisitions, manually or with the TACS, as such, the quality of the reconstructed images from the 4D respiratory CBCT acquisitions is limited in the proof-of-concept implementation demonstrated in this study. Image quality improvements could be facilitated by additional vendor support. Overall, the concepts introduced in this study of (1) varying gantry rotation velocity and (2) combing/considering motion compensated imaging reconstruction techniques to facilitate 4D respiratory CBCT on robotic imaging systems for interventional procedures are indeed generalisable to other imaging systems and situations. However, to overcome the key limitations identified within the current study primarily requires additional vendor support, which is non-trivial to receive, and thus presently restricts the generalisability and replicability of the study beyond the current experimental setup.

A novel reconstruction workflow was developed for this study to address the unique challenges arising from the acquisition and phantom design. Due to the fast gantry rotation, phase correlated tomography would be extremely sparse and limited angle without implementing some degree of data sharing between phases. The phantom had low contrast relative to a typical patient, and we believe this resulted in image blur when iterative reconstruction algorithms were used. Our workflow starts with a 3D FDK reconstruction as that uses all acquired data to produce a sharp image with the exception of motion blur at moving anatomy. The 3D FDK volume initializes the ROOSTER reconstruction which we found greatly accelerated convergence, we believe because the 3D FDK volume has largely correct anatomy which restricts the null space of the limited tomography model. The resulting ROOSTER images still contain some blur and with low contrast, but sufficient for coarse deformable image registration and inferring a motion model. That motion model is then used for MCFDK reconstruction as the MCFDK uses all acquired data when reconstructing each phase and produce sharp images even when underlying contrast is low, largely addressing the acquisition and phantom challenges.

Finally, another challenge faced by interventional thoracic CBCT acquisitions is metal artifacts arising from procedural hardware (e.g., ablation probes and biopsy needles). An emerging approach to minimizing the effect of metal artifacts arising from procedural hardware is to take advantage of the flexibility of modern robotic CBCT imaging systems and implement non-circular imaging trajectories [41]. Previous investigations focusing on orthopedic and neurological interventions have identified and implemented non-circular imaging trajectories on robotic CBCT imaging systems to reduce metal artifacts around spine stabilization [42] and hip replacement [43] hardware. Although it is beyond the scope of the work presented here, in the future there is potential to combine non-circular CBCT imaging trajectories designed to reduce metal artifacts with 4D respiratory CBCT acquisitions to provide combined metal and motion artifact free CBCT images in the interventional room.

## Conclusion

Overall, the current implementation of 4D respiratory CBCT explored in this study with various gantry velocities combined with motion compensated algorithms improved image sharpness for the slower gantry rotations considered (2°/s and 10°/s) compared to conventional acquisitions over a variety of patient traces.In the future, it is proposed that more advanced motion compensated algorithms be investigated and applied to the conventional acquisition, allowing 4D information to be derived from free-breathing 3D acquisitions.

## Data Availability

The concept and information presented in this paper are based on research and is not commercially available. Due to regulatory reasons its future availability cannot be guaranteed.
